# Recombinant GII.P16/GII.4 Sydney 2012 Was the Dominant Norovirus Identified in Australia and New Zealand in 2017

**DOI:** 10.3390/v10100548

**Published:** 2018-10-09

**Authors:** Jennifer H. Lun, Joanne Hewitt, Grace J. H. Yan, Daniel Enosi Tuipulotu, William D. Rawlinson, Peter A. White

**Affiliations:** 1 School of Biotechnology and Biomolecular Sciences, Faculty of Science, University of New South Wales, Sydney 2052, NSW, Australia; j.lun@unsw.edu.au (J.H.L.); grace.j.yan@unsw.edu.au (G.J.H.Y.); d.enosi@unsw.edu.au (D.E.T.); w.rawlinson@unsw.edu.au (W.D.R.); 2 Institute of Environmental Science and Research, Kenepuru Science Centre, Porirua 5022, New Zealand; joanne.hewitt@esr.cri.nz; 3 SAViD (Serology and Virology Division) Department of Microbiology, Prince of Wales Hospital, Sydney 2031, NSW, Australia; 4 School of Medical Sciences, Faculty of Medicine, University of New South Wales, Sydney 2052, NSW, Australia; 5 School of Women’s and Children’s Health, Faculty of Medicine, University of New South Wales, Sydney 2052, NSW, Australia

**Keywords:** norovirus, recombinant, molecular epidemiology, next generation sequencing, MiSeq, clinical, wastewater, genetic diversity, Australia, New Zealand

## Abstract

For the past two decades, norovirus pandemic variants have emerged every 3–5 years, and dominate until they are replaced by alternate strains. However, this scenario changed in 2016 with the co-circulation of six prevalent viruses, three of which possessed the pandemic GII.4 Sydney 2012 capsid. An increased number of institutional gastroenteritis outbreaks were reported within the Oceania region in mid-2017. This study identified emerging noroviruses circulating in Australia and New Zealand in 2017 to assess the changing dynamics of the virus infection. RT-PCR-based methods, next generation sequencing, and phylogenetic analyses were used to genotype noroviruses from both clinical and wastewater samples. Antigenic changes were observed between the capsid of pandemic Sydney 2012 variant and the two new Sydney recombinant viruses. The combination of these antigenic changes and the acquisition of a new ORF1 through recombination could both facilitate their ongoing persistence in the population. Overall, an increased prevalence of GII.P16/GII.4 Sydney 2012 viruses was observed in 2017, replacing the GII.P16/GII.2 recombinant that dominated in the region at the end of 2016. This shift in strain dominance was also observed in wastewater samples, demonstrating the reliability of wastewater as a molecular surveillance tool.

## 1. Introduction

Acute gastroenteritis (AGE) is the second most common human infectious disease, and results in 1.4 million deaths worldwide each year [1]. Norovirus is now the leading cause of viral AGE in all age groups globally, and is estimated to cause 677 million cases each year, accounting for ~210,000 deaths [2]. Despite the self-limiting nature of the disease, severe and prolonged symptoms can be observed in children, the elderly, and in immunocompromised individuals [3]. Norovirus is highly transmissible due to (i) the low infectious dose [4], (ii) virus stability within the environment, and (iii) continued virus shedding after symptoms have resolved. Therefore, outbreaks of norovirus-associated gastroenteritis frequently occur in semi-closed environments including hospitals, aged-care facilities, childcare centers, and cruise ships.

Norovirus is a genus of genetically-diverse, single-stranded, RNA viruses within the *Caliciviridae* family. Based on full-length amino acid capsid (VP1) sequences, norovirus can be tentatively divided into seven genogroups (GI-GVII) and more than 40 genotypes [5,6,7]. The viruses GI, GII, and GIV are associated with human infections; however, GII viruses are responsible for the majority of human norovirus infections. 

Recombination (antigenic shift) and antigenic drift are the two main mechanisms that drive norovirus evolution [8]. Antigenic drift is caused by amino acid changes within the capsid protein (VP1), primarily within the protruding domain (P2), which enables the virus to escape from population host immunity [9]. The capsid of GII.4 noroviruses continuously undergo epochal evolution, resulting in the emergence of new pandemic variants approximately every 3–5 years [3,10,11,12,13]. Recombination usually occurs near the ORF1/ORF2 overlap, which enables the exchange of the entire structural or non-structural regions [14], leading to the creation of novel viruses, some of which also have pandemic potential [15]. 

For the past two decades, the pandemic GII.4 variants usually account for 60%–80% of all norovirus infections, and dominate until another variant emerges [3]. However, a change in norovirus molecular epidemiology has been observed over the past three years. In 2016, we saw a decline of the pandemic Sydney 2012 variant, concomitant with the emergence of two novel GII.4 recombinant viruses, both of which retained the Sydney 2012 capsid but acquired new non-structural regions (GII.P4 New Orleans 2009/GII.4 Sydney 2012 and GII.P16/GII.4 Sydney 2012) [16]. The emergence of GII.P4 New Orleans 2009/GII.4 Sydney 2012 was first detected in 2013, and was created through a recombination event between the Sydney 2012 pandemic variant with its pandemic predecessor GII.4 New Orleans 2009 variant [17]. Following its identification, this recombinant virus was also identified in Australia, New Zealand [16,18], Denmark [19], England [20], and South Africa [21]. The other novel GII.4 recombinant, GII.P16/GII.4 Sydney 2012, was first identified in the Oceania region in mid-2015, and was found circulating at a low prevalence in South Korea, Germany, and Japan in 2016 [16,22,23,24]. In mid-late 2016, an increase of a third novel recombinant, GII.P16/GII.2, was observed in Australia and New Zealand [16]. This virus was also detected around the globe in 2016, including in Japan [25], the United States [26], China (51% of outbreaks) [27], and Europe [24,28], where it was responsible for 14%–42% of all norovirus outbreaks.

Therefore, continuous surveillance of circulating norovirus strains at a population level is essential for early identification of novel viruses which may have pandemic potential. In this study, we compared the noroviruses which were circulating in Australia and New Zealand in 2017, assessed the changing dynamics of epidemic variants, and identified emergent norovirus variants which arose from recombination and antigenic variation. Molecular epidemiology of norovirus is commonly conducted using clinical samples collected from symptomatic patients, which is not representative of all circulating noroviruses in a population. Consequently, the second aim of this study was to compare the norovirus GII genotype distribution between wastewater and clinical samples.

## 2. Materials and Methods

### 2.1. Collection of Clinical Specimens

All norovirus-positive clinical specimens were collected as part of routine diagnostic services or norovirus surveillance between January and December 2017. Multiplex reverse transcription polymerase chain reaction (RT-PCR) and norovirus lateral flow enzyme immunoassay (EIA) were used during the routine diagnostic services for norovirus detection. The study was approved by the University of New South Wales (UNSW) Human Research Ethics Advisory Panel (HREAP) (HC12221, HC16826 and HC17459). For Australia, 243 specimens were collected via the New South Wales (NSW) Ministry of Health from gastroenteritis institutional outbreaks and sporadic cases. For New Zealand, representative specimens from 238 separate norovirus outbreaks in 2017 were collected.

### 2.2. Collection of Wastewater Samples

Monthly influent samples (250 mL) were collected from Bondi and Malabar wastewater treatment plants (WWTP) between January and December 2017. Melbourne samples were collected from Werribee, western WWTP, between May and December 2017. All samples were stored at −80 °C on the day of collection.

### 2.3. Viral Concentration and RNA Extraction

Stool suspensions (10–20% in water) were prepared from clinical specimens, followed by viral RNA extraction, as described previously [13,29]. Ultracentrifugation was used to concentrate viruses in wastewater samples prior to viral RNA extraction, as described in [16]. Frozen aliquots of MS2 bacteriophage, with a concentration of 2.6 × 10^6^ ± 1.6 × 10^5^ genome copies/20 μL, were used as process control to validate RNA extraction and RT-PCR amplification [16].

### 2.4. Reverse Transcription PCR (RT-PCR) Amplification and Sequencing

For Australian clinical specimens, a norovirus GI and GII duplex RT-PCR was performed targeting the 5′ end of capsid gene for norovirus genotyping [11], along with a norovirus GI or GII RT-PCR targeting the ORF1/ORF2 overlap for the identification of potential recombinants [29,30]. For New Zealand clinical specimens, RT-PCR was conducted targeting region B of the RdRp, region C of the capsid, and across the ORF1/ORF2 overlap [29]. All RT-PCR products were Sanger sequenced and genotyped using phylogenetic analysis [16].

For wastewater samples, the 5′ end of norovirus GII capsid was amplified, followed by a second round PCR for the addition of universal sequencing adapters, following the manufacturer’s protocol (Illumina, San Diego, CA, USA). PCR amplicons were purified using AMPure XP beads (Beckman Coulter, Brea, CA, USA) prior to next generation sequencing (NGS) library preparation. NGS libraries were prepared and sequenced on the Illumina MiSeq platform, as described previously [16]. Library fragment sizes were evaluated on a Tape Station D1000 (Agilent Technologies, Santa Clara, CA, USA) prior to sequencing. Full-length capsid genes of representative GII.4 were amplified, as described previously [15], and Sanger sequenced.

### 2.5. Norovirus Phylogenetic Analysis

Partial polymerase (GI-171 bp, GII-172 bp) and partial capsid (GI-295 bp, GII-282 bp) sequences were used for phylogenetic analyses to determine genotype of norovirus-positive samples and confirmed using an online genotyping tool (http://www.rivm.nl/mpf/norovirus/typingtool). MUSCLE was used to align the GI and GII sequences and phylogenetic trees were constructed using the maximum likelihood method [31]. 

### 2.6. NGS Data Analysis

The software package Geneious, v 9.1.7 (Biomatters, Auckland, New Zealand) was used to analyze MiSeq data. Raw data were filtered to retain sequencing reads between 200 to 400 nt in length. Paired-end sequences were merged and mapped to a list of GII references (*n* = 88) using Geneious mapper with medium sensitivity and default parameters. The proportion of each GII genotype was calculated, as described in [16]. Subsequently, all GII.4 reads were mapped to GII.4 variant sequences (*n* = 3) to determine the abundance of individual GII.4 variants in the population. 

### 2.7. Analysis of Amino Acid Variation within GII.4 Capsid Sequences

Full length GII.4 capsid (VP1) protein sequences were aligned and compared with reference sequences obtained from GenBank. Variable informative sites were analyzed and determined by the website DIVEIN (http://indra.mullins.microbiol.washington.edu/DIVEIN/) [32]. Informative sites are identified when the same amino acid mutation at the same position is shared by at least two strains. 

### 2.8. Molecular Adaptation Analysis of the Norovirus GII.4 Capsid

To investigate the likelihood of positive selection within the GII.4 capsid, 42 representative sequences were retrospectively selected between the period 2014 to 2017 and compared to reference sequences obtained from NCBI. The web server (http://datamonkey.org/) [33] was used to identify potential positive and negative selection within the capsid coding sequence. The three codon-based methods used were Mixed Effect Model of Evolution (MEME), Fixed Effects Likelihood (FEL), and Fast, Unconstrained Bayesian AppRoximation (FUBAR) [11]. The significance threshold was set to *p* value of 0.07 for both MEME and FEL, and posterior possibility of 0.9 for FUBAR. 

## 3. Results

### 3.1. Gastroenteritis Outbreak Increase in 2017

**Figure 1:**
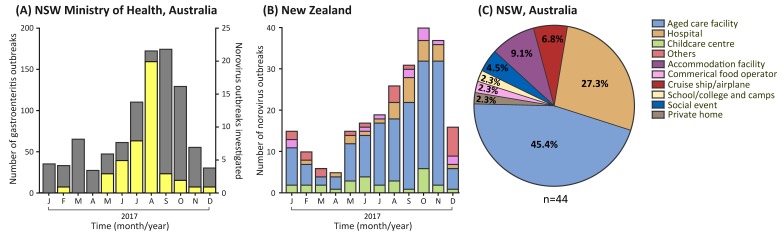
**The number of gastroenteritis and norovirus outbreaks reported in the Oceania region, 2017.** (**A**) The monthly number of institutional gastroenteritis outbreaks reported to the NSW Ministry of Health department in 2017 (highlighted in grey), and the number of norovirus-associated outbreaks investigated in this study (highlighted in yellow); (**B**) The number of norovirus outbreaks reported to New Zealand Ministry of Health in 2017. The outbreak settings are categorized as indicated by the legend; (**C**) A total of 44 norovirus outbreaks were identified in NSW, Australia, throughout this study period. The outbreak settings are represented by different colors in the legend.

In August 2017, a sudden increase of gastroenteritis outbreaks was reported to the NSW Ministry of Health, Australia, peaking in September 2017 (*n* = 175 outbreaks). This represented a 4.8-fold and 2.7-fold increase compared to the average number of monthly outbreaks detected in 2015 and 2016, respectively (Figure 1A) [16]. Additionally, a rise in norovirus outbreaks was also observed in New Zealand, with the peak occurring in October 2017 (*n* = 42 outbreaks) (Figure 1B). This was the highest number of norovirus outbreaks recorded in New Zealand since the emergence of the Sydney 2012 pandemic strain (Figure 1B), and a 2.7-fold rise when compared to the previous two years [16]. Therefore, we aimed to determine if the increased norovirus outbreaks were caused by the emergence of a novel norovirus. 

### 3.2. Outbreak Settings

A total of 282 norovirus outbreaks (Australia = 44 and New Zealand = 238) were investigated from samples collected during 2017. Of those, GI was identified as the etiological agent in 52 outbreaks (18.4%), whilst GII was the causative agent in 228 outbreaks (80.9%). The remaining two outbreaks identified in New Zealand were caused by a mix of GI and GII noroviruses. In Australia, most outbreaks occurred in aged care facilities (45.4%), followed by hospitals (27.3%), accommodation facilities (9.1%), cruise ships/airplanes (6.8%), and social events (4.5%), with the remaining detected in three other settings (Figure 1C). Aged care facilities were also identified as the most common outbreak setting in New Zealand (63.5%), followed by childcare centers (12.2%), hospitals (10.9%), commercial food operators (5.0%), school/colleges (2.9%), and others (Figure 1B). 

### 3.3. Circulating GI Noroviruses

**Figure 2:**
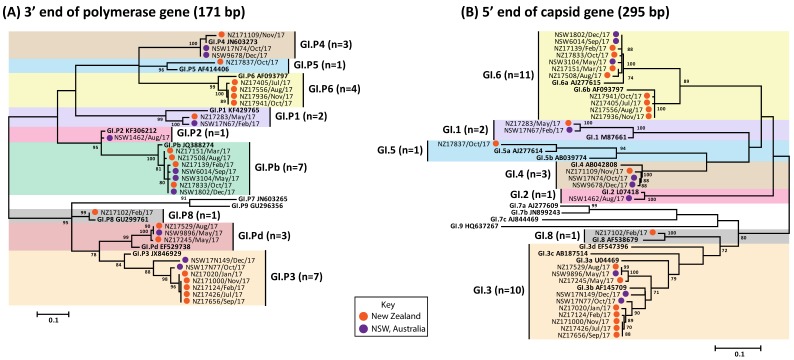
**Phylogenetic analysis of polymerase (RdRp) and capsid (VP1) regions of GI noroviruses.** Representative norovirus GI strains isolated in this study (*n* = 29), from both NSW, Australia and New Zealand, are shown in this phylogenetic analysis. They are denoted with a colored bullet (•), where Australian samples are represented in purple and New Zealand samples in orange. All samples are labelled with their geographical location and time of strain isolation. Reference strains were obtained from the GenBank database, labelled with their genotype and accession number. (**A**) Maximum likelihood phylogeny derived from partial 3′ end of polymerase gene (171 bp) of GI noroviruses. (**B**). Maximum phylogeny derived from partial 5′ end of capsid gene (295 bp) of GI noroviruses. Sequence alignments were performed with MUSCLE algorithm. Maximum likelihood phylogenetic trees were produced using MEGA 5 software (https://www.megasoftware.net/) with bootstrapping test of 1000 replicates, based on the Kimura 2-parameter model. The bootstrap percentage values are shown at each branch point for values ≥70%. The number of substitutions per site is indicated by the scale bar.

**Figure 3:**
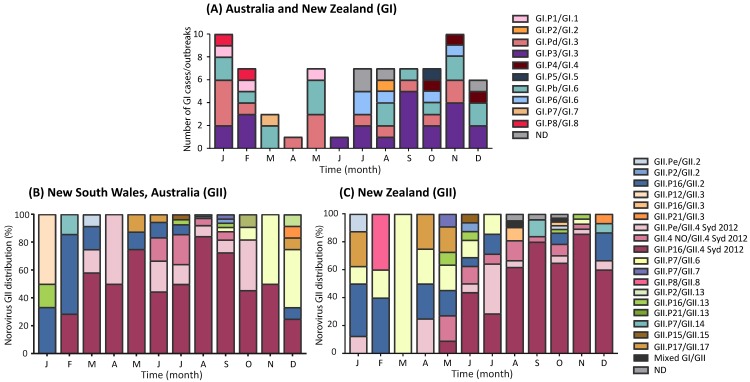
**Monthly distribution of norovirus genotypes identified in the Oceania Region in 2017.** (**A**) The number of GI norovirus genotypes identified in the Oceania region (Australia and New Zealand) during the study period. A total of 73 samples were collected and identified as GI norovirus, 52 cases were linked to outbreaks and the remaining were sporadic cases (*n* = 21). Both polymerase and capsid regions were sequenced to determine its genotype, with each genotype denoted by different colors as indicated in the legend. The ND represents samples that had incomplete genotyping results where only the capsid or the polymerase region was determined. (**B**) The monthly genotype distribution of GII noroviruses identified in New South Wales, Australia, throughout the study was examined. A total of 220 GII cases were identified in this study, where 44 were linked to outbreaks and the remaining were considered sporadic cases (*n* = 176). (**C**) All samples collected from New Zealand were of outbreak samples and a total of 184 GII outbreaks were investigated in 2017. The monthly genotype distribution of GII noroviruses are shown.

GI represented 9.1% (*n* = 22/243) of all noroviruses sequenced in Australia and phylogenetic analysis identified a total of five capsid genotypes (Figure 2). GI norovirus was found to be more prevalent in New Zealand than Australia, where it was responsible for 21.4% (*n* = 51/238) of all norovirus outbreaks, with six capsid genotypes identified (Figure 2). From the 73 GI noroviruses isolated from Australia and New Zealand, ten GI strains were identified, of which GI.P3/GI.3 (30.1%, 22/73) was the most predominant, followed by GI.Pb/GI.6 (21.9%, 16/73) and GI.Pd/GI.3 (20.5%, 15/73) (Figure 3A).

### 3.4. Circulating GII Noroviruses

**Figure 4:**
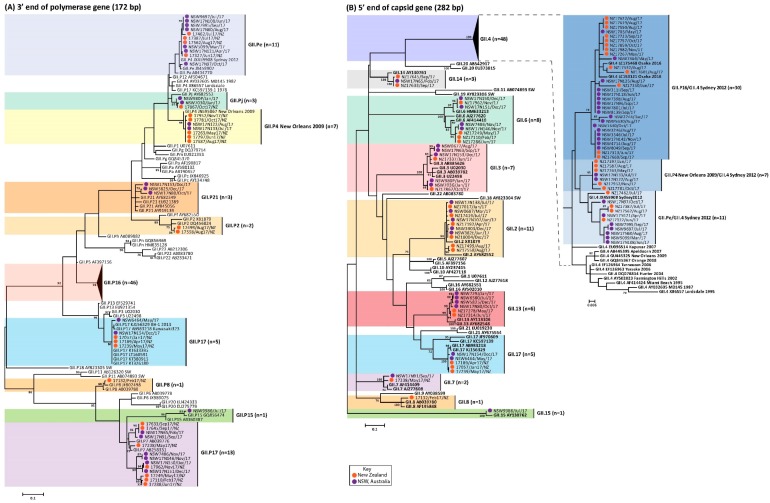
**Phylogenetic analysis of polymerase (RdRp) and capsid (VP1) regions of GII norovirus.** Representative norovirus GII strains isolated in this study (*n* = 92) from both NSW, Australia and New Zealand, are shown in this phylogenetic analysis. They are denoted with a colored bullet (•), where Australian samples are represented in purple and New Zealand samples in orange. All samples are labelled with their geographical location and time of strain isolation. Due to the number of sequences grouped within GII.P16 and GII.4, the sequences within those clusters were compressed and represented by the black triangles. Reference strains were obtained from the GenBank database, labelled with their genotype and accession number. (**A**) Maximum likelihood phylogeny derived from partial 3′ end of polymerase gene (172 bp) of GII noroviruses. (**B**). Maximum phylogeny derived from partial 5′ end of capsid gene (282 bp) of GII noroviruses. Sequence alignments were performed with MUSCLE algorithm. Maximum likelihood phylogenetic trees were produced using MEGA 5 software with bootstrapping test of 1000 replicates, based on the Kimura 2-parameter model. The bootstrap percentage values are shown at each branch point for values ≥70%. The number of substitutions per site is indicated by the scale bar.

**Figure 5:**
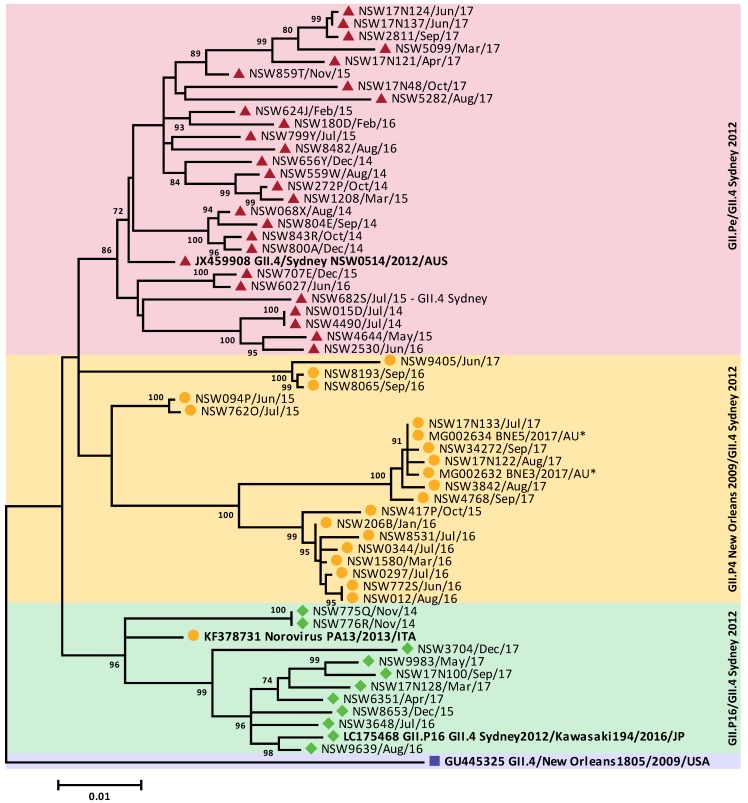
**Phylogenetic analysis of GII.4 full length capsid sequences.** Representative full-length GII.4 capsid sequences were selected between 2013 to 2017 and compared to prototype GII.4 pandemic variants and GII.4 recombinant viruses. Sample sequences are denoted by the sample location, sample ID and date of collection. Prototype sequences of each GII.4 pandemic variant and GII.4 recombinant viruses are indicated in bold. Reference sequences were obtained from GenBank, labelled with its accession number and country of isolation. The GII.Pe/GII.4 Sydney 2012 sequences are denoted with a red triangle, GII.P4 New Orleans 2009/GII.4 Sydney 2012 with a yellow circle, the GII.P16/GII.4 Sydney 2012 sequences with a green diamond and the prototype GII.4 New Orleans 2009 is denoted by a purple square. The asterisks (*) denote sequences obtained from the Bruggink et al. 2018 study [34]. Sequence alignments were performed with MUSCLE algorithm. Maximum likelihood phylogenetic trees were produced using MEGA 5 software with bootstrapping test of 1000 replicates, based on the Kimura 2-parameter model. The bootstrap percentage values are shown at each branch point for values ≥70%. The scale bar indicates the number of substitutions per site.

Norovirus GII was identified as the most predominant genogroup, responsible for 84.8% (*n* = 408/481) of all cases/outbreaks investigated in 2017 from Australia and New Zealand (Figure 3B,C and Figure 4). Based on the phylogenetic analyses (Figure 4), interestingly, the GII.4 capsid sequences segregated into three different clusters, allowing differentiation of the GII.4 capsid containing viruses (Figure 4B and Figure 5). In January 2017, the recombinant GII.P16/GII.2 was dominant in New Zealand (37.5%) and Australia (33.3%); however, its prevalence slowly declined, which was concomitant with the increase of the recombinant GII.P16/GII.4 Sydney 2012 in the region (Figure 3B,C). In 2017, the GII.P16/GII.4 Sydney 2012 recombinant was the dominant strain responsible for 56.8% (*n* = 138/243) and 42.9% (*n* = 102/238) of all cases/outbreaks identified in both Australia and New Zealand, respectively. In Australia, the prevalence of GII.P16/GII.4 Sydney 2012 reached to 75.0% of total cases/outbreaks in May and then to 84.2% by August 2017 (Figure 3B), whilst in New Zealand, the virus caused >60% of norovirus GII outbreaks between June and December (Figure 3B).

In Australia, the second most predominant GII norovirus identified was the pandemic Sydney 2012 variant (10.8%, *n* = 24/221) after the GII.P16/GII.4 Sydney 2012, followed by the recombinant GII.P4 New Orleans 2009/GII.4 Sydney 2012 (6.8%, *n* = 15/221), GII.P16/GII.2 (6.3%, *n* = 14/221) and GII.P7/GII.6 (5.0%, *n* = 11/221) (Figure 3B and Figure 4). However, in New Zealand, GII.P16/GII.2 was found to be the second most predominant GII virus identified (9.1%, *n* = 17/187), after the most dominant GII.P16/GII.4 Sydney 2012. This was followed by GII.Pe/GII.4 Sydney 2012, GII.P4 New Orleans 2009/GII.4 Sydney 2012 and GII.P7/GII.6, each accounted for 7.0% of GII outbreaks (*n* = 13/187) (Figure 3C and Figure 4). 

### 3.5. Antigenic Variation within the GII.4 Capsids

**Figure 6:**
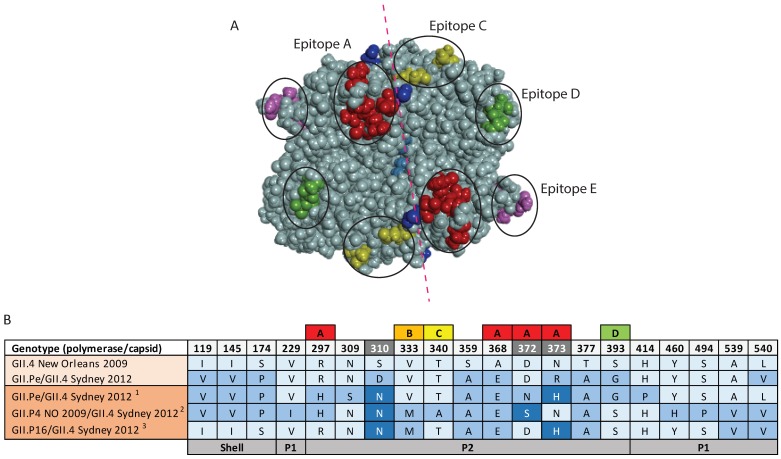
**Capsid residue variation and antigenic variation within the full-length capsid of GII.4 recombinant viruses.** Full-length capsid sequence of novel GII.4 recombinant viruses were collected between July 2014 and December 2017. Consensus sequences of contemporary GII.Pe/GII.4 Sydney 2012^1^ (*n* = 20), GII.4 New Orleans 2009/GII.4 Sydney 2012^2^ (*n* = 13) and GII.P16/GII.4 Sydney 2012^3^ (*n* = 9) were generated and compared to pandemic variants (New Orleans 2009 and Sydney 2012) for the identification of antigenic variations, especially within the P2 protruding domain. (**A**) Characterized blockade antibody epitopes are highlighted in the different colors; epitope A (red), epitope C (yellow), epitope D (green) and epitope E (purple). The residues within P2 domain, especially those within epitope A, which differed from the pandemic Sydney 2012 variant. The red dotted line shows symmetry. (**B**) Antigenic variations observed between full length capsid sequences of pandemic Sydney variant and the new Sydney 2012 recombinants. Labelled boxes above the antigenic sites indicate sites within known blockade epitopes A–D that are important determinants of viral antigenicity. The A–D epitopes identified in GII.4 capsid are colored; epitope A (red), epitope B (orange), epitope C (yellow) and epitope D (green). The numbers across the top panel indicate the amino acid position within the VP1 sequence, hypervariable sites with ≥3 amino acids substitutions across all sequences are shaded in grey. Residues that vary from the GII.4 New Orleans 2009 sequence over time are indicated by shades of blue. The bottom panel indicates the positions of the shell, P1 and P2 domains within the VP1 capsid protein. The prototype of each pandemic/recombinant variants are indicated in the lightest shade of orange.

The multiple recombination events of the Sydney 2012 pandemic variant is unusual, and has facilitated viruses with this capsid to persist around the globe. Full-length GII.4 amino acid capsid consensus sequences of the three Sydney 2012 viruses were examined to identify antigenic variation and evidence of positive selection, especially within putative antigenic sites (epitopes A–E) and histo-blood group antigen (HBGA) binding pocket. Representative capsid sequences of GII.Pe/GII.4 Sydney 2012 (*n* = 20), GII.P4 New Orleans 2009/GII.4 Sydney 2012 (*n* = 13) and GII.P16/GII.4 Sydney 2012 (*n* = 9), were used to generate consensus sequences. Compared to the original pandemic Sydney capsid, the consensus sequences of the contemporary Sydney 2012 viruses varied at a number of residues (Figure 6). Firstly, the consensus sequence of GII.Pe/GII.4 Sydney 2012 varied at seven residues, including residues 297, 372, and 373 in epitope A, residue 310 in the NERK motif, and residues located in the P2 domain (309) and P1 domain (414 and 540) (Figure 6). Secondly, the GII.P4 New Orleans 2009/GII.4 Sydney 2012 capsid consensus sequence varied at 11 residues, in which six were located within the main antigenic epitopes; epitope A (297, 372 and 373), B (333), C (340) and D (393). The remaining changes were observed in P1 domain (residue 229, 460, 494, 539) and NERK motif (310) (Figure 6). Finally, the GII.P16/GII.4 Sydney 2012 recombinant strain varied at ten residues located in; epitope A (373), epitope B (333), epitope D (393), NERK motif (310), shell domain (119, 145, 174), P1 domain (539, 540) and P2 domain (377) (Figure 6). Of all variable sites identified, residue 373 was found to be under positive selection by all three codon-based methods (MEME, FEL and FUBAR). 

### 3.6. Norovirus GII Genotype Distribution in Wastewater Samples

**Figure 7:**
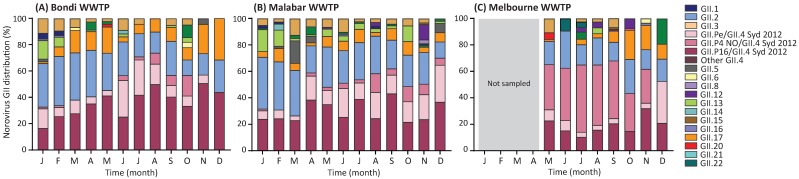
**Norovirus GII genotype distribution in wastewater samples collected from Sydney and Melbourne, 2017.** The norovirus GII capsid genotypic distribution was determined in wastewater samples by amplicon sequencing using NGS technology. After amplification of the capsid region, a second round PCR was performed for the addition of adapters prior NGS library preparation. Libraries were sequenced on the MiSeq platform and an average of 152,879 reads were generated for each sample. Geneious was used for merging and mapping of the reads to the norovirus GII reference sequences. (**A**) The monthly genotype distribution of norovirus GII viruses in wastewater samples collected from Bondi WWTP in Sydney, Australia. (**B**) Norovirus GII genotypic diversity was examined in wastewater samples collected monthly from Malabar WWTP in Sydney, Australia. (**C**) The monthly norovirus GII genotype diversity was also examined in Melbourne, all samples were collected from western wastewater treatment plant. Samples were not collected between January and April 2017. Norovirus GII capsid genotype and GII.4 recombinants are labelled in different colors as indicated by the legend.

The use of wastewater samples for viral surveillance can enhance the surveillance of norovirus at a population scale [16,35]. Partial GII norovirus capsid regions were amplified from monthly samples collected from three WWTP sites from Sydney and Melbourne and sequenced on the Illumina MiSeq platform. A total of 4,892,127 reads were generated from 32 wastewater samples, with an average of 152,879 reads per sample. Across the three sites, 16 capsid genotypes were identified; the dominant capsid genotypes included GII.4 (52.6%), GII.2 (24.5%), GII.3 (9.8%), GII.17 (5.8%) and GII.13 (5.5%) (Figure 7). 

GII.2 was found to be the predominant capsid genotype at the Bondi and Malabar WWTPs in January (33.1% and 39.1% of reads, respectively) and February (37.4% and 36.4%, respectively). However, a steady decline of GII.2 was observed for the remainder of the year at both sites, accompanied by an increase of GII.4 viruses around March 2017 (Figure 7A,B). Subtle but distinct differences can be found between the three GII.4 Sydney capsid sequences, which enabled the inference of polymerase genotype. At the Bondi WWTP, an increase in GII.P16/GII.4 Sydney 2012 recombinant was observed; 25.6% in February, 41.3% in May, 41.9% in July, and reached its highest levels in November 2017 (50.7%) (Figure 7A). At the Malabar WWTP, an average of 23.8% of GII.P16/GII.4 reads were seen in the first three months of 2017, then 39.1% in July, 43.3% in September, and 36.8% in December (Figure 7B). This virus was the most predominant GII strain from July to December, and accounted for an average of 37.5% of the monthly genotype distribution for 2017 in Sydney (Figure 7A,B). 

For the Melbourne WWTP, GII.4 was the dominant capsid genotype between May and December 2017 (average of 60.9% of monthly reads) (Figure 7C). Surprisingly, the recombinant GII.P16/GII.4 Sydney 2012 was not the predominant strain in Melbourne, with a monthly average of 19.1% of reads across 2017. Of the three GII.4 recombinants, GII.P4 New Orleans 2009/GII.4 Sydney 2012 was the most prevalent virus in wastewater samples, constituting 34.1% of reads in May, 45.5% in August, and 25.7% in November. GII.2 viruses were identified as another dominant genotype throughout the study period in Melbourne, accounting for a monthly average of 19.1% (Figure 7C).

## 4. Discussion

A total of six pandemic norovirus variants have emerged in the past two decades, all of which may be classified within the GII.4 lineage [11], with the most recent emerging in 2012, namely Sydney 2012 (GII.Pe/GII.4 Sydney 2012) [15,36]. Recently Sydney 2012 has undergone recombination with both its predecessor pandemic strain (New Orleans 2009) and a GII.P16 virus to create two new strains (GII.P4 New Orleans 2009/GII.4 Sydney 2012 and GII.P16/GII.4 Sydney 2012), with reported potential to evade population immunity and cause outbreaks [16,26,28,37,38]. 

In the winter months of 2017, an increase in gastroenteritis outbreaks was observed in Australia and New Zealand when compared to the previous two years (Figure 1). This increase is consistent with the study by Bruggink et al., 2018 in Victoria, Australia, where the number of outbreaks in 2017 doubled in July (*n* = 22) compared to May 2017 (*n* = 11) [34]. Overall, this data suggested the continued dominance of GII.P16/GII.2 or the emergence of a novel norovirus. Therefore, we genotyped circulating noroviruses within the Oceania region in 2017. The prevalence of GII.P16/GII.2 diminished in early 2017, and was replaced by GII.P16/GII.4 Sydney 2012, which was the dominant virus in both Australia (57.0%) and New Zealand (43.2%) for the remainder of the year. This replacement suggests that a GII.4 capsid is one essential requirement for norovirus persistence within the population. A previous study by Parra et al. showed non-GII.4 viruses were genetically more static [39], and only minor divergence was observed within the GII.2 capsid sequence over 40 years [40]. This lack of variance in GII.2 antigenic domains could explain their short-lived duration. In contrast, GII.4 viruses have the highest rate of evolution (5.4 × 10^−3^ nucleotide substitutions/site/year) compared to other genotypes, signifying their ability to facilitate the emergence of new variants, and as shown in this study, their ability to replace GII.2 as a dominant strain [39,41]. 

The GII.P16/GII.4 Sydney 2012 recombinant retained the original Sydney 2012 capsid, albeit with slight modifications. Based on the increased circulation of this strain, the change in the non-structural region may also have contributed to immune escape and conferred higher virological fitness [38,42]. This is supported by the cocirculation and increased prevalence of GII.P16/GII.4 Sydney 2012 and GII.P16/GII.2 viruses in 2016/17, both of which possess the GII.P16 polymerase [16]. Indeed GII.P16/GII.2 could represent the “stepping stone” precursor to GII.P16/GII.4 Sydney 2012 virus. This hypothesis is supported by Tohma et al., who showed the polymerase of GII.P16/GII.4 Sydney 2012 was derived from the GII.P16/GII.2 virus [40]. In addition, alterations within the non-structural genes may allow viral persistence through escape of the cytotoxic T lymphocyte response of the host, which has been demonstrated for both hepatitis C virus and human immunodeficiency virus infections [43,44], although this is not well studied for norovirus.

No definitive pandemic variant has emerged since 2012; instead, two new recombinants have maintained a GII.4 Sydney 2012 capsid [17,23]. This suggests that antigenic drift, where point mutations occurred within the capsid P2 domain, may have contributed to immune escape [8]. Therefore, consensus GII.4 capsid sequences collected in Oceania between 2014 and 2017 were used to identify potential sites of antigenic drift and positive selection within Sydney 2012 capsids. Of the blockade epitopes (A–E), epitope A is believed to be one of the most important determinants of antigenic change [45] and associated with the loss of blockade antibody binding, as well as the emergence of new GII.4 variants [45,46]. In this study, significant positive selection was detected at residue 373 of epitope A in all three recombinant GII.4 Sydney 2012 strains, consistent with previous findings [11,42]. 

Epitope D contains the histo-blood group antigen (HBGA) carbohydrate binding sites [9,47], and changes in residue 393 showed loss of human monoclonal antibody binding and modulation in Lewis A and B binding [9,45,46], and therefore, affect attachment and entry of the virus. Within epitope D, amino acid reversion to sequence found in the New Orleans 2009 capsid at residue 393 (epitope D), was identified in both the GII.P4 New Orleans 2009/GII.4 Sydney 2012 and GII.P16/GII.4 Sydney 2012 recombinant viruses. Additionally, residues 119, 145, and 174 within shell domain of GII.P16/GII.4 Sydney 2012 also reverted to residues found in the previous pandemic variant, New Orleans 2009. Even though antigenic reversion is commonly identified in viruses [48,49,50,51], the benefits are not well understood. However, previous studies have proposed that reversion is a result of immune escape [52], and it can limit the antigenic repertoire [53]. 

Additional antigenic changes were also observed after 2014, with all three strains with the Sydney 2012 capsid possessing an asparagine (N) amino acid at residue 310, as opposed to the aspartic acid (D) found in the original in Sydney 2012 variant. Residue 310 is located within the NERK motif, which regulates capsid structural conformation, and thus antigenic changes may result in less or inaccessible epitopes [42]. This persistence of Sydney 2012 capsid was probably enhanced by the sudden emergence of multiple recombinant noroviruses, with different ORF1 regions, but all containing the Sydney 2012 capsid. This acquisition of a novel non-structural region together with the observed capsid amino acid changes likely increased the epidemiological fitness of the recombinant viruses through immune escape. Therefore, the combination of both recombination and antigenic variation influenced the continued circulation of the GII.4 Sydney capsid.

Consistent with our clinical data, a high abundance of GII.P16/GII.4 Sydney 2012 was detected in wastewater collected from Sydney and a switch in strain predominance was observed in March (Bondi) and April (Malabar), where GII.P16/GII.4 Sydney 2012 capsid replaced the previously dominant GII.2 capsid genotype. In contrast to Sydney wastewater, no change in capsid genotype dominance was observed in Melbourne WWTP, and GII.4 viruses persisted as the dominant capsid genotype throughout the study period. This could be due to the lack of sampling in the early months of 2017, where the switch was observed in Bondi and Malabar WWTP. Furthermore, the recombinant GII.P4 New Orleans 2009/GII.4 Sydney 2012 was identified as the dominant GII virus in Melbourne WWTP. This result correlated with the study performed by Bruggink et al. in Victoria, Australia, in which GII.P4 New Orleans 2009/GII.4 Sydney 2012 was shown to be responsible for 69% of all outbreaks investigated between January and September 2017 [34], demonstrating the usefulness of wastewater samples for norovirus surveillance. 

Molecular epidemiological surveillance of norovirus is essential to identify new circulating recombinants and emerging strains at a population level, particularly when outbreak activity escalates. Moreover, knowledge of circulating noroviruses is useful for effective vaccine design. Norovirus surveillance in Australia and New Zealand, 2017, showed an increase of GII.P16/GII.4 Sydney 2012 viruses, concomitant with the decrease of the 2016 prevalent strain GII.P16/GII.2. Our study highlights the importance of norovirus recombination, together with antigenic capsid changes for the emergence of new epidemic strains. With the use of NGS technologies, the switch in GII norovirus dominance was also observed in wastewater at a population level in Sydney, and the dominant New Orleans 2009/GII.4 Sydney 2012 strain also was identified in Melbourne. This highlights the reliability of wastewater as a norovirus surveillance tool.
